# Malignant epithelioid angiomyolipoma of the submandibular gland: A case report

**DOI:** 10.1016/j.radcr.2024.09.027

**Published:** 2024-09-24

**Authors:** Ayesha Afzal, Muhammad Junaid Tahir, Asma Asghar, Islah Ud Din, Muhammad Atif Naveed

**Affiliations:** Department of Radiology, Shaukat Khanum Memorial Cancer Hospital and Research Center, Lahore, Punjab province, Pakistan

**Keywords:** PEComa, Salivary gland, Angiomyolipoma, Metastasis

## Abstract

An epithelioid angiomyolipoma (a perivascular epithelioid cell tumor) is a rare mesenchymal neoplasm with distinctive cellular morphology and nonspecific imaging appearances. Mostly reported perivascular epithelioid cell tumors (PEComas) are benign; however, rarely, PEComas can be malignant with pulmonary, hepatic, nodal, and osseous metastases. We present a case of a 40-year-old man with malignant right submandibular salivary gland PEComa, metastasized to the bones, lungs, and liver. We are going to discuss the diagnosis and management options of the rare disease of metastatic PEComa of the submandibular salivary gland.

## Introduction

Angiomyolipomas constitute a category of mesenchymal neoplasms, distinguished by an amalgamation of aberrant blood vessels, spindle cells, and mature adipocytes. It is known to arise from the perivascular epithelioid cells (PEC) [1]. Perivascular epithelioid cell tumors (PEComas) encompass diverse entities within the family of mesenchymal tumors stemming from PECs, including angiomyolipomas and pulmonary lymphangioleiomyomatosis [2,3].

PEComas typically exhibit a higher incidence in females, as PEComas of the female genital tract account for nearly 25% of PEComas of all body sites [4]. PEComas are known to manifest across a wide age range. These tumors have been observed in nearly every part of the body over the past few decades. Histologically, they are characterized by the presence of epithelioid cells arranged in a perivascular pattern, featuring clear to pale eosinophilic granular cytoplasm and centrally located round-to-oval nuclei with inconspicuous nucleoli [5]. Although the perivascular distribution of tumor cells is a hallmark, it may not always be evident, with most PEComas showing a delicate vascular stroma surrounding tumor nests. Immunohistochemically, the characteristic markers include co-expression of melanocytic (HMB-45 and/or Melan-A) and myoid markers [6].

The growing interest in PEComas has led to an increasing number of reports detailing their presence in various anatomical locations There are several case reports of this disease involving the head and neck region. In this report, we are going to discuss the behavior and course of PEComa arising in the submandibular salivary gland.

## Case presentation

**Fig. 1:**
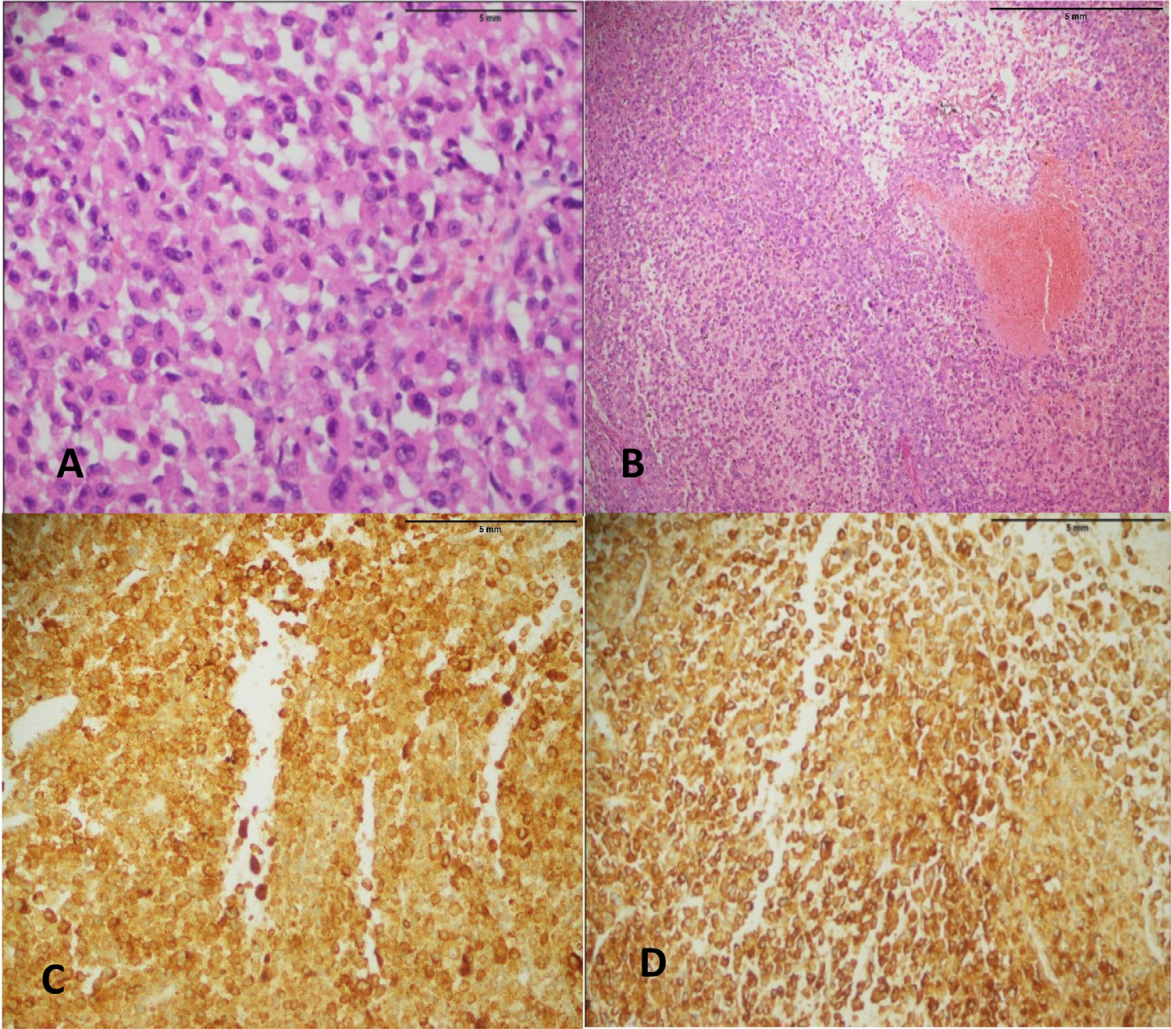
A and B: Histopathological study showing sheeted arrangement of neoplastic cells with abundant clear to granular eosinophilic cytoplasm, moderate nuclear atypia, and some with prominent nucleoli. (HE stains - A: 40X and B: 10X). C and D: Immunohistochemical stains are positive for cathepsin K and HMB 45 (40X).

Our case is of a 40-year-old man who presented in outpatient clinic with slowly growing lump in the right submandibular region for the last 6 months. The patient had already undergone excisional biopsy at an outside hospital facility 3 weeks back and no prior imaging was available for review. On physical examination, an excisional biopsy scar was seen in the right submandibular region with no palpable neck lymph node. The histopathology revealed poorly differentiated malignant neoplasm with giant cells showing a sheeted arrangement with abundant clear to granular eosinophilic cytoplasm, moderate nuclear atypia, and some with prominent nucleoli. A battery of immunohistochemical stains were applied. HMB-45, cathepsin K, melan-A, and TFE3 were found to be positive, which raised the possibility of a malignant perivascular epithelioid tumor (PEComa) (Fig. 1).

**Fig. 2:**
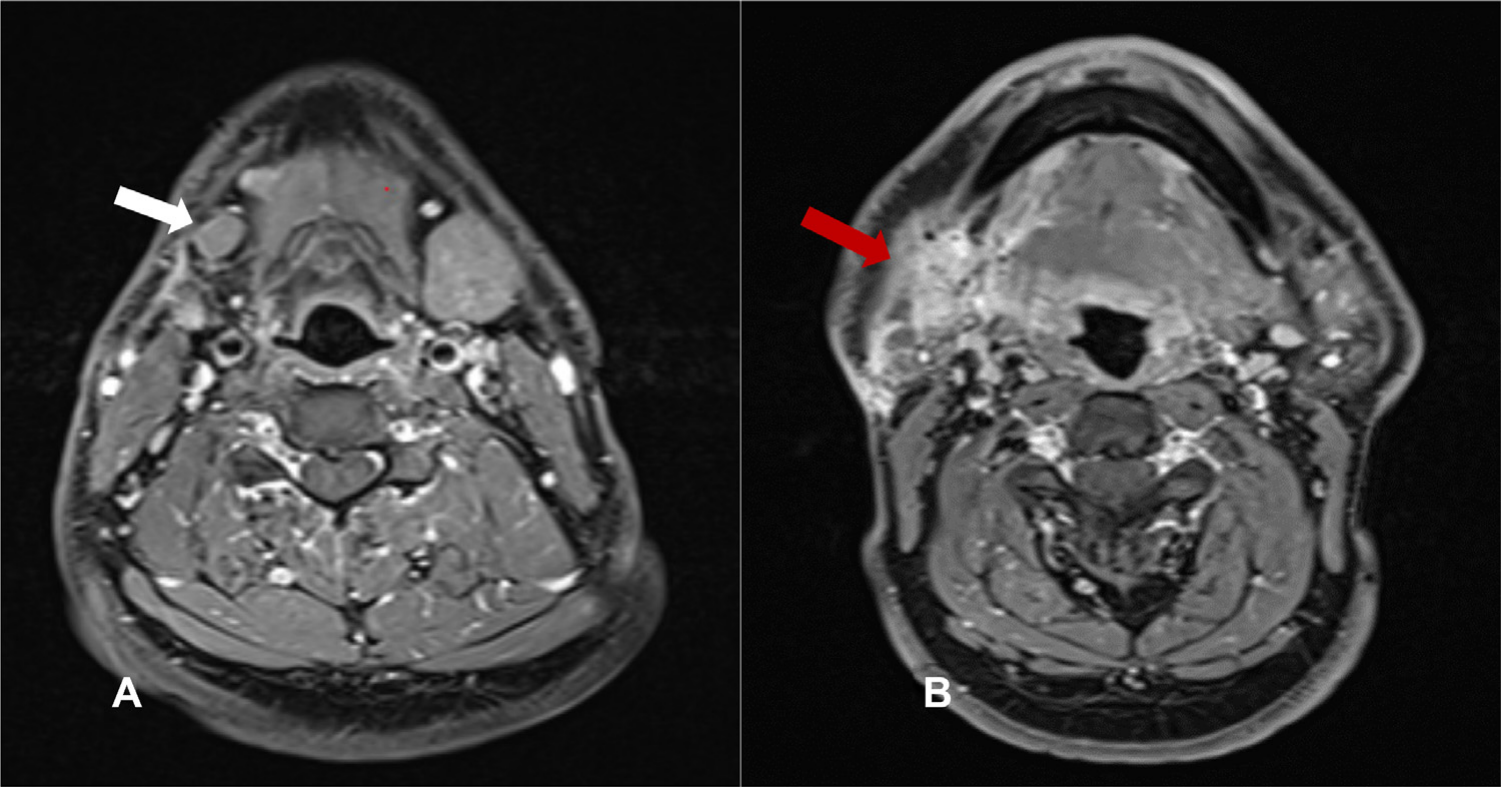
Axial T1 weighted post gadolinium MR images of the neck with fat saturation at the level of submandibular region showing an enlarged right submandibular lymph node (white arrow on Fig. 3A) with diffuse heterogeneous enhancement and fat stranding (red arrow on Fig. 3B) in the right submandibular region, suggesting granulation tissue and scar formation.

**Fig. 3:**
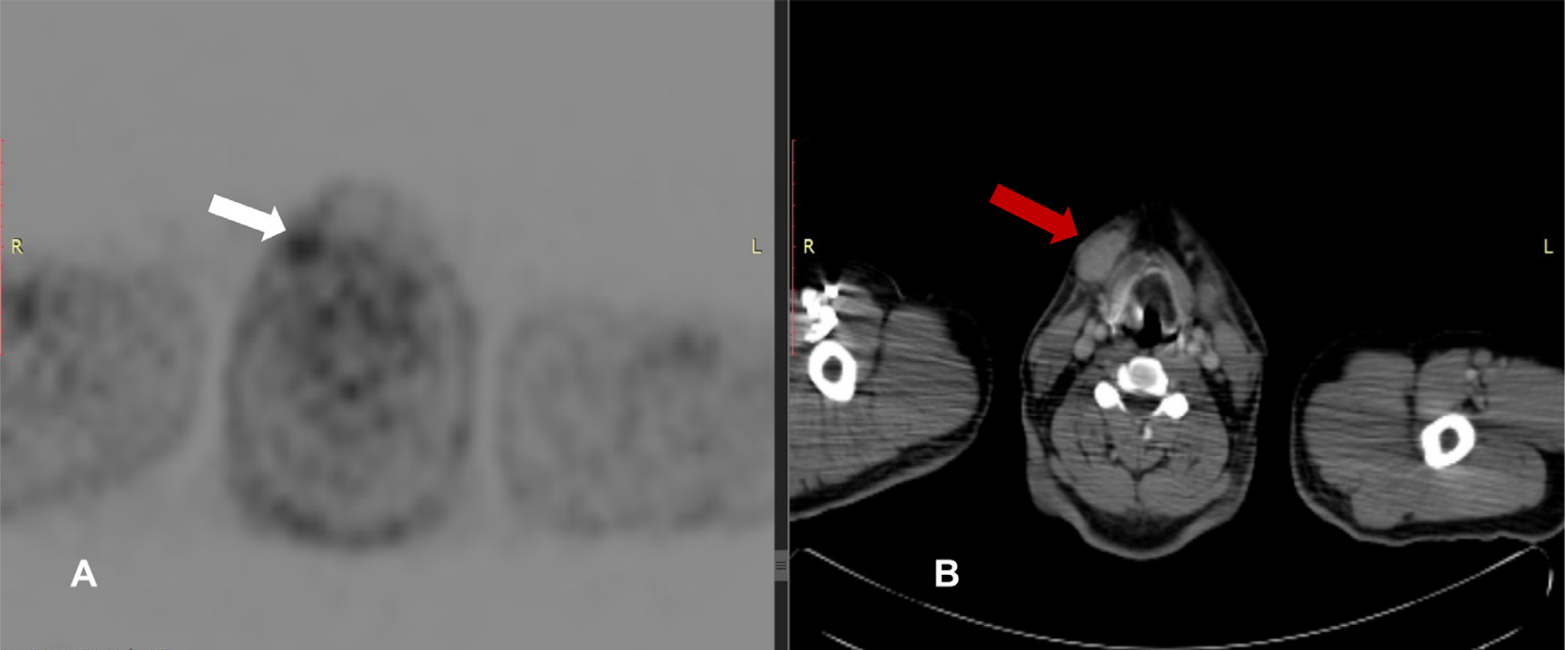
(A) Axial attenuation correction FDG PET image at the level of submandibular region shows a hypermetabolic lymph node SUV 2.3. (B) Axial CT component of the study at the same level reveals enlarged right submandibular lymph node.

MRI face and neck was performed to evaluate for the residual disease after surgery. It showed scarring and granulation tissue in the right submandibular region (Fig. 2). The right submandibular lymph node measured 14 × 9 mm and was considered enlarged. Further, mild FDG uptake of 2.3 SUV was detected in this lymph node on PET imaging (Fig. 3).

This node was subjected to ultrasound-guided fine needle aspiration and perivascular epithelioid tumor metastases was proven on cytological analysis. Right neck dissection was performed, followed by radiotherapy to the right neck.

**Fig. 4:**
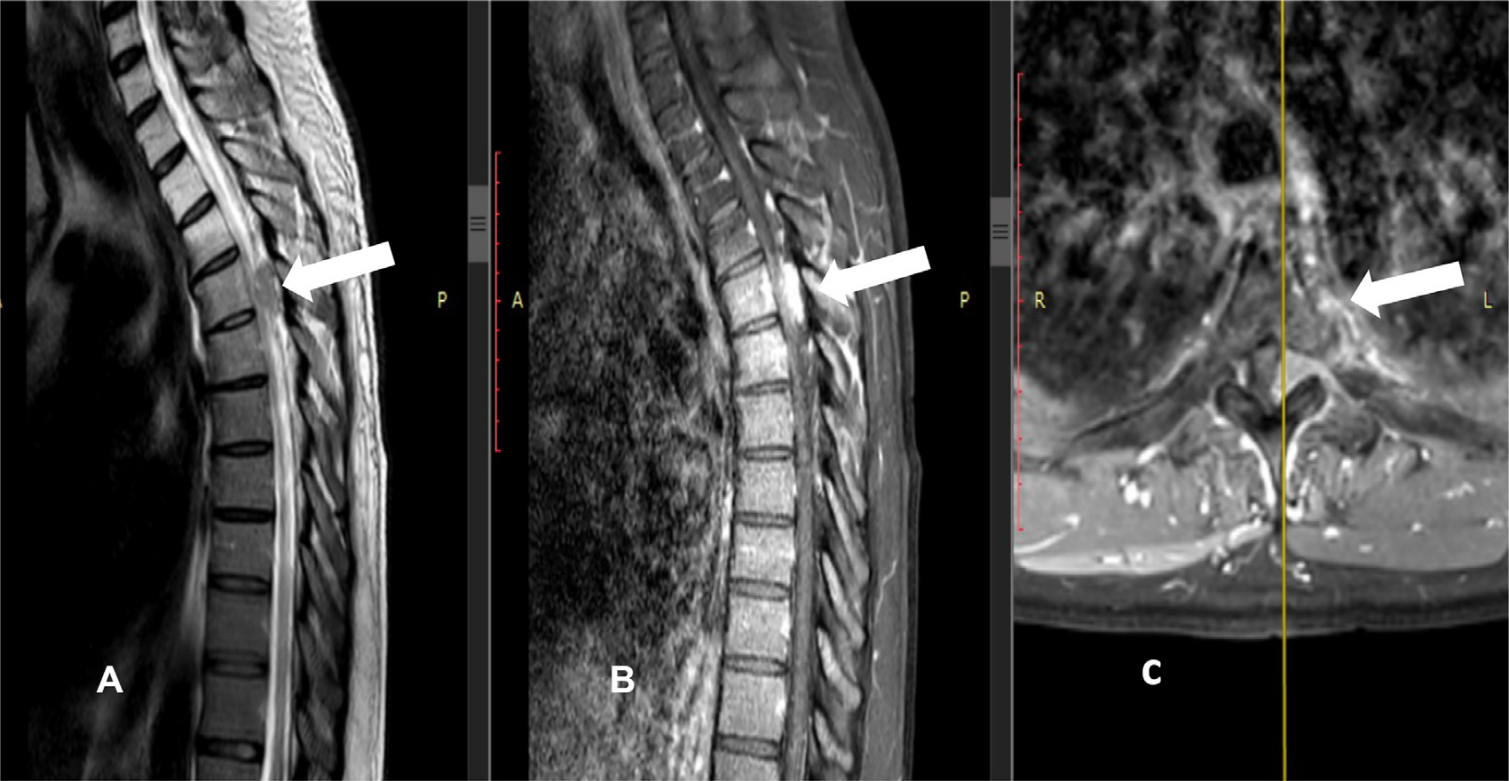
Sagittal T2 weighted (A), T1 weighted MR images following gadolinium administration with fat saturation in sagittal (B) and axial (C) planes show a dural-based lesion at level of T4 vertebra, causing spinal canal stenosis and cord compression (epidural spinal cord compression scale [ESCC] grade 3 cord compression).

The patient developed osseous metastasis after almost 10 months, with dural-based lesion at T4, causing severe spinal canal stenosis and cord compression, epidural spinal cord compression scale (ESCC) 3 (Fig. 4). There was also left L2 nerve root impingement due to extraosseous soft tissue component, causing foraminal stenosis. Subsequently, a decompressive L2 laminectomy was performed.

The contrast enhanced staging CT was performed 5 months later. It showed right submandibular region disease recurrence with new pulmonary and hepatic metastases. Systemic treatment started with palliative intent.

**Fig. 5:**
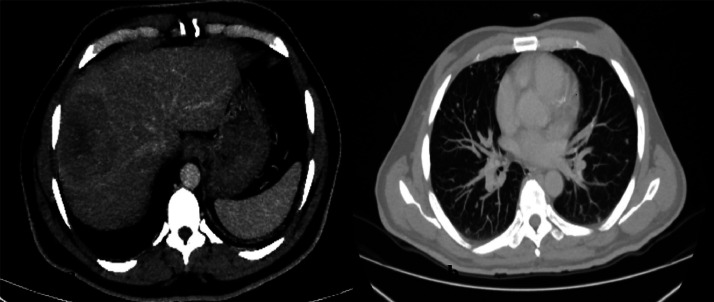
**(A)** Axial CT image of the abdomen at the level of the liver after intravenous iodinated contrast administration reveals a heterogeneously hypodense hepatic lesion in right lobe of liver, segment 8, consistent with metastasis**. (B)** Axial CT image of the chest shows multiple scattered subcentimetric soft tissue nodules in both lungs, consistent with metastasis.

Despite chemotherapy, pulmonary, hepatic, and spinal metastases had progressed. The right submandibular soft tissue showed fullness and mass like thickening with enhancement which suggested disease recurrence. Bilateral scattered pulmonary nodules and heterogeneously hypodense hepatic lesions (Fig. 5) were suggestive of metastases. Thus, due to an extensive disease burden, growing ascites, and hepatic decompensation, the patient succumbed to death.

## Discussion

Our case provides a valuable insight into the clinical course and management of malignant PEComas, particularly those arising in uncommon locations such as the submandibular salivary gland. PEComas, a subgroup of mesenchymal tumors characterized by their distinctive histopathological features and variable clinical behavior, pose significant diagnostic and therapeutic challenges.

The initial observation of perivascular epithelioid cells (PEC) dates to 1943 when Apitz [7] identified them in renal angiomyolipomas. The term “PEComa” was coined in 1996 by Zamboni to describe a tumor originating in the pancreas [8]. Since then, these tumors have been documented in various organs, although our case represents one of the earliest reported occurrences in the salivary gland. Most PECs express markers associated with myogenic and melanocytic lineage, such as HMB-45, HMSA-1, MelanA/mart1, microphthalmia transcription factor (MITF), actin, and desmin [3] The cytokeratin and S-100 protein are usually absent [9]. In our malignant PEComa case, positive immunoreactivity was observed for HMB-45 and cathepsin K.

Angiomyolipoma (AML), lymphangioleiomyomatosis, clear cell “sugar” tumor of the lung (CCST), clear cell myomelanocytic tumor of the falciform ligament/ligamentum teres (CCMMT), and unusual clear tumors of the pancreas, rectum, uterus, and vulva are all included in the PEComa family [6,10]. PEComa in the submandibular region with hepatic, lung, and osseous metastases with a 3-year clinical course has rarely been documented in the literature.

Folpe et al. [11] established the criteria for determining malignant potential of PEComas, encompassing parameters such as size exceeding 5 cm, a mitotic rate exceeding 1/50 hpf, necrosis, high nuclear grade and cellularity, vascular invasion, and an infiltrative growth pattern. Tumors lacking these criteria are typically deemed benign, while those meeting fewer than 2 criteria are categorized as having uncertain malignancy potential. Conversely, tumors exhibiting 2 or more high-risk features, as seen in our case, are classified as malignant. This can be translated to our case as the patient had arrangement of neoplastic cells in sheets with abundant clear to granular eosinophilic cytoplasm, moderate nuclear atypia, and some with prominent nucleoli, suggestive of malignant potential. However, these criteria remain somewhat contentious due to the rarity of this tumor type and inconsistent follow-up data. In 2012, Bleeker et al. [12] revisited these criteria, analyzing new data, and found that the rate of “aggressive behavior” in tumors classified as malignant was lower than initially proposed, at 51% compared to the original 71%. Consequently, a revised set of Folpe criteria was proposed, which now focuses solely on tumor size exceeding 5 cm and a high mitotic rate.

Surgical resection represents the cornerstone of treatment for localized disease, with adjuvant radiotherapy or chemotherapy in select cases [1]. In our patient, postoperative imaging revealed heterogeneous enhancement, mass-like thickening and fat stranding in the surgical bed, suggestive of residual disease with postoperative changes. The subsequent development of spinal metastasis with spinal cord compression underscores the aggressive nature of malignant PEComas and the importance of MR imaging.

As MRI and CT technologies progress, there is an increasing recognition of their utility in diagnosis. Nevertheless, PEComas manifest a broad range of imaging characteristics, and the literature lacks comprehensive descriptions of radiological features. MRI stands out for its superior tissue contrast and specificity, enabling more accurate identification of lipomatous elements, specific to the liver, and assisting in PEComa diagnosis as in our case, heterogeneously hypodense hepatic lesions were identified which were suggestive of metastasis. Prior studies have suggested that hypervascularity and arteriovenous features in contrast-enhanced CT or MRI serve as valuable diagnostic indicators of PEComa [13,14].

The most frequent sites of PEComas are the uterus and retroperitoneum, though the tumor can be found virtually anywhere [15]. The present case represents malignant PEComa that arises in the submandibular gland. This is the first case that has been reported in the submandibular gland to the best of our knowledge.

While our case underscores the aggressive nature of malignant PEComas and the challenges associated with disease management, it also highlights the need for further research to elucidate the underlying molecular mechanisms driving tumor progression. Future studies exploring novel therapeutic targets and treatment strategies are warranted to improve outcomes for patients afflicted with this rare and aggressive malignancy.

## Conclusion

The presented case contributes to the existing literature on malignant PEComas, emphasizing the importance of comprehensive diagnostic evaluation, multimodal therapy, and vigilant surveillance in optimizing patient outcomes. The occurrence of PEComa in the submandibular region is exceedingly rare, and its prognosis varies depending on histological characteristics. Further research is required to advance our understanding of this rare entity and to identify more effective therapeutic interventions.

## Authors’ contributions

A.A. (Ayesha Afzal) and M.A.N conceived and designed the case report, and were responsible for data collection and acquisition of data. A.A. (Ayesha Afzal) and M.J.T. performed the literature review and wrote the manuscript. M.A.N., I.U.D., M.J.T. and A.A. (Asma Asghar) reviewed and critically revised the manuscript. All authors have approved the final manuscript.

## Patient consent

This case report was conducted with the approval of the institutional review board (EX-04-08-23-02) and written informed consent for the publication of this case report was obtained from the patient's wife. All patient data were de-identified to maintain confidentiality.
